# Prevention of birth defects in the pre-conception period: knowledge and practice of health care professionals (nurses and doctors) in a city of Southern Brazil 

**Published:** 2015-10

**Authors:** Flávia Romariz Ferreira, Heloisa Regina Russo Akiba, Edward Araujo Júnior, Elisabeth Niglio Figueiredo, Anelise Riedel Abrahão

**Affiliations:** 1 *Department of Administration and Public Health, Paulista School of Nursing, Federal University of São Paulo (EPE-UNIFESP), São Paulo-SP, Brazil.*; 2 *Department of Obstetrics, Paulista School of Medicine, São Paulo Federal University (EPM-UNIFESP), São Paulo-SP, Brazil.*

**Keywords:** Congenital defects, Public health, Primary prevention

## Abstract

**Background:**

Some congenital defects can be prevented in the pregestational stage. However, many health professionals are not prepared to provide counselling to couples regarding the same.

**Objective:**

This study aimed to assess the performance of doctors and nurses from a primary health-care unit in Florianopolis, Brazil, in preventing birth defects in the preconception period based on the recommendations of the Control Center of Disease Prevention.

**Materials and Methods:**

This descriptive cross sectional study was performed at a tertiary referral center. In this study, a semi-structured questionnaire was provided to 160 health professionals comprising doctors and nurses who were actively involved in providing primary health care in family health programs. The non-parametric Chi-square (χ^2^) test was used to analyse the data obtained through multiple choice questions**.**

**Results:**

Our results showed that although 81.9% of health professionals provided health-care assistance based on protocols, and only 46.2% professionals were aware of the presence of the topic in the protocol. Of the recommendations provided by the Control Center of Disease Prevention, the use of folic acid was the most prescribed. However, this prescription was not statistically different between nurses and doctors (P=0.85).

**Conclusion:**

This study identified the fragile nature in these professional’s knowledge about the prevention of birth defects in pre-conception period, as evidenced by the inconsistency in their responses.

## Introduction

The Brazilian Ministry of Health has made family planning an integral part of health-care practices focusing on women, men, or couples, with a vision of global and integral health-care services. This service guarantees care from conception to contraception during the entire cycle in all the service networks (1).

Until the 1970s, public policies aimed at women’s health were focussed on their reproductive function and were limited to pregnancy and childbirth, with an emphasis on women as mothers, constituting the mother-child-like model (2). However, the concept of family planning should not only be restricted to procreative aspects, but also should include needs and aspirations of a family, such as housing, food, schooling and leisure (3). However, to this day, the concept of family planning remains associated with reproductive aspects and involves activities involving birth control.

Pregestational care is a basic-level prevention, involving both a woman and her partner; thus, it promotes the health of both the genders (4-6). To highlight such affirmation, the Control Center of Disease Prevention (CDC) states that all women, men and couples should be encouraged to plan their reproductive lives. In this way, a health professional is responsible for informing a couple of the best tools to be used for their reproductive objectives by respecting the couple’s culture, religion and literacy level (4). Pregestational care is important to prevent congenital defects. The main components of pregestational care include risk identification, health-related education and medical and psychosocial intervention (7, 8).

The practice of health professionals who work in the Family Health Strategy (FHS) programme, a model aimed at providing primary attention, should be uniform in a community so that it allows better detection and control of risks to the mother and foetus. Thus, it is important that all the recommendations of the CDC should be adequately disseminated to provide systematic help to this group (9).

Congenital defects are an important indicator of public health because they are among the 10 leading causes of infant morbidity and mortality (10). Moreover, there is a direct relationship between maternal and foetal health and early access to and quality of specialised health services. Therefore, it is very important to promote public health actions for improving and expanding prenatal care for pregnant women who are carrying foetuses with congenital anomalies (11). Congenital defects have diverse aetiologies, and many of these factors can be prevented at a primary level from early preconception to postconception, however the causes of congenital defects are unknown in almost 50% of cases (12).

This research aims to describe the performance of doctors and nurses from a basic health-care unit in the municipality of Florianopolis, Brazil, in preventing congenital defects in the pregestational period. The research aims to (a) design a profile of doctors and nurses that work in the public health system in the municipality of Florianopolis, (b) identify the use of help protocols for preventing congenital defects during pregestational care, (c) identify the actions taken by doctors and nurses for preventing congenital defects during pregestational care and (d) determine the interest of these health professionals regarding this theme.

## Materials and methods

This study was first submitted to and approved by the Health Research Project Follow-up Committee at the Health County Secretary of Florianopolis, Brazil, and by the Ethics Committee in Research on Human Beings from the Federal University of São Paulo (UNIFESP). This descriptive cross-sectional study was performed from January to April 2009 and involved 160 health professionals out of 194 doctors and nurses from Florianopolis’ basic health-care units. Thirty-four health professionals were excluded from the study as they were absent at the time of data collection because of vacation or medical leave. All the participants included in this research signed informed consent forms.

Data were collected through questionnaires designed from information provided by the CDC and from questions taken from Tom Heyes, Sarah Long, and Nigel Mathers questionnaires, which were applied to recognise the attitudes and beliefs regarding the prevention of congenital anomalies in the pregestational period (13, 14).

The questionnaire that designed for this study included 11 questions, of which 10 were multiple choice questions and 1 was an open-ended question. The questionnaire was filled directly by health professionals who visited the primary health attention unit. Answering the open-ended question required some knowledge of the theme and identification of intervention related to the theme; this question was called ‘non-stimulated’ question by the authors. All the multiple choice questions were called ‘stimulated’ questions because they had choices that allowed the health professionals to recollect the subject matter. Before the initiation of this research, the designed questionnaire was given to 12 health professionals from a primary health attention unit belonging to an administration in the southeast zone of São Paulo, Brazil. This allowed the researchers to identify difficulties associated with answering such a questionnaire and suggestions to improve the same. 

The stimulated questions were designed to (a) test the knowledge of regional health professionals with respect to the pertinent practices used for preventing congenital defects during the pregestational period, (b) verify whether health professionals know at whom the pregestational care should be aimed at and (c) test the consistency among answers provided by the health professionals by crosschecking distinct questions related to practices and interventions that prevent congenital defects during the pregestational period. The non-stimulated question was designed to understand the intervention that the regional health professionals prescribed and when they prescribed it to prevent congenital defects. Because it was an open-ended question, the interviewed professionals were able to offer an explanation about their practice without any interference.

**Statistical analysis**

Data obtained from the multiple choice questions were inserted in a table and were analysed in MS Excel 2007 (Microsoft Corp., Redmond, WA, USA). The absolute and relative frequencies of the obtained answers were presented in five groups: (a) characteristics of these health professionals, (b) knowledge of these health professionals for preventing congenital defects in the pregestational period, (c) attitude of these professionals for preventing congenital defects in the pregestational period, (d) interest and perception of these health professionals about the theme and (e) veracity and agreement among the responses. To analyse the data obtained through the stimulated questions, a non-parametric Chi-square (χ2) test was used. As far as the open-ended questions are concerned, the data was handled separately to better understand the information exposed by the professionals through the Content Analysis proposed by Bardin, from which categories expressed by absolute and relative frequencies have emerged (15).

## Results

**Characteristics of the health professionals**

Of the 194 professionals from 46 basic health units present in the municipality of Florianopolis, Brazil, 82.5% (n=160) professionals satisfied the inclusion criteria and answered the questionnaire. Of these 160 professionals, 89 were nurses and 71 were doctors (Table I).

**Knowledge of the health professionals in preventing congenital defects in the pregestational period**

The Secretary of Health of the Florianopolis municipality has presented a specific protocol geared towards women’s health (16). However, the presence of this protocol was not specified in the study questionnaire so as to not limit the professionals' answers. This decision enabled the answers to be exclusively based on their professional practice.

**Table I:** Characteristics of nurses and doctors from the family heath strategy in the municipality of Florianopolis, Brazil, in 2009

**Variable**	**Nurses n (%)**	**Doctors n (%)**	**Total n (%)**
**Age (years)**			
20–29	39 (43.8)	15 (21.1)	54 (33.8)
30–39	31 (34.8)	33 (46.5)	64 (40.0)
40–49	17 (19.1)	10 (14.1)	27 (16.9)
≥50	2 (2.2)	13 (18.3)	15 (9.4)
**Gender**			
Female	82 (92.1)	39 (54.9)	121 (75.6)
Male	7 (7.9)	32 (45.1)	39 (24.4)
**Graduate study**			
Public health	27 (30.3)	30 (42.3)	57 (35.6)
No specification	15 (16.9)	20 (28.2)	35 (21.9)
Others	20 (22.5)	10 (14.1)	30 (18.8)
Ongoing	7 (7.9)	2 (2.8)	9 (5.6)
Only undergraduate	20 (22.5)	9 (12.7)	29 (18.1)
**Time after graduation(years)** *			
0–5	36 (40.4)	11 (15.5)	47 (29.3)
5–10	24 (27.0)	31 (43.7)	55 (34.4)
10–20	19 (21.3)	11 (15.5)	30 (18.7)
20–30	8 (9.0)	14 (19.7)	22 (13.7)
≥30	2 (2.2)	4 (5.6)	6 (3.7)
**Experience (years)** **			
0–1	11 (12.3)	5 (7.0)	16 (10.0)
1–5	40 (45.0)	25 (35.2)	65 (40.6)
5–10	29 (32.5)	26 (36.6)	55 (34.4)
≥10	9 (10.1)	15 (21.1)	24 (15.0)

* Time after graduation: number of year after medical or nurse degree.

** Experience: number of years in the clinical practice

Of the 160 professionals, 81.9% (n=131) mentioned that their practice was based on a pre-established protocol while 1.9% (n=3) mentioned that they were unaware whether their practices were associated to any protocol. In all, 74 (46.2%) professionals were aware of the theme in the given protocol, 63 (39.4%) professionals mentioned that the theme under discussion was not a part of any given protocol, and 23 (14.4%) professionals were unaware of the theme.

Among all the participants, 63.7% (n=102) mentioned that they took actions to prevent congenital anomalies during pregestational care and 16.9% (n=27) mentioned that they did not follow any practice for the same. As far as the recognition of the actions taken in relation to the prevention of the congenital anomalies in pre-gestational care, out of the 63.7% (n=102) of the professionals who affirmed having carried out some action related to prevention, was observed that the action was well below expectation.

As far as the more frequent intervention between the two studied professional groups is concerned as shown in Table II, the use of folic acid was the most mentioned, representing 54.4% (n=87) of the findings, 46.1% (n=41) of which represented nurses’ conduct and 64.8% (n=46) that of doctors, however without significant differences (P=0.85)

**Attitudes of the professionals regarding the issue**

The attitudes of the professionals according to the open-ended question aimed at preventing congenital defects in the pregestational period are shown in table III. The most cited intervention (19.4%) was family investigation, which was mentioned by 28.2% of doctors and 12.4% of nurses. This was followed by solicitation of pre-pregnancy examinations, which accounted for 18.7% of the total interventions.

Interventions practised and mentioned in the open-ended question showed no statistically significant differences between the two professional groups (p=0.43). With respect to the participation of doctors and nurses from the FHS in training for the prevention of congenital defects in the pregestational care, it was noted that 137 (85.6%) professionals claimed that they did not receive any training for preventing these anomalies. Of the 160 professionals, only 4 nurses and 15 doctors confirmed to have participated in some training.

**Table II:** Interventions recommended by the control center of disease prevention for preventing congenital defects during pregestational care, categorised according to nurses and doctors` practice in the family health strategy in the municipality of Florianopolis, Brazil, in 2009

**Intervention practice recommended by CDC**	**Nurses (n = 89)**	**Doctors (n = 71)**	**Total (N = 160)**
	**n (%)**	**n (%)**	**n (%)**
Folic acid in the pregestational period	41 (46.5)	46 (64.8)	87 (54.4)
Alcohol, drugs, or smoking	29 (32.6)	22 (31.0)	51 (31.9)
Special care with medicine (accutane, oral anti-clotting, and anti-epileptic medication)	20 (22.5)	22 (31.0)	42 (26.2)
Vaccination (rubella and hepatitis)	15 (16.8)	9 (12.7)	24 (15.0)
HIV/DST special care	7 (7.9)	7 (9.9)	14 (8.7)
Diabetes, hypertension, hypothyroidism control	3 (3.4)	3 (4.2)	6 (3.7)
Obesity	1 (1.1)	1 (1.1)	2 (1.3)

CDC: Control Center of Disease Prevention

Chi-square (χ2) test; P = 0.85

**Table III:** Interventions for preventing congenital defects during pregestational care that are not recommended by the CDC, according to the practice performance of nurses and doctors from the Family Health Strategy in the municipality of Florianopolis, Brazil, in 2009

**Interventions**	**Nurses**	**Doctors**	**Total**
	**n (%)**	**n (%)**	**n (%)**
Family investigation	11 (12.4)	20 (28.2)	31 (19.4)
Solicitation of pre-pregnancy exams/medical appointment	15 (16.8)	15 (21.1)	30 (18.7)
Guidance of a balanced diet	11 (12.4)	10 (14.1)	21 (13.1)
Pre-pregnancy guidance	9 (10.1)	2 (2.8)	11 (6.9)
Investigation of exposure to teratogen-based agents	4 (4.5)	6 (08.4)	10 (6.2)
Pap examination consultation and breast cancer prevention	6 (6.7)	2 (02.8)	8 (5.0)
Physical activity guidance	4 (4.5)	2 (02.8)	6 (3.7)
Investigation on occupational hazard	3 (3.4)	0 (00.0)	3 (0.19)
Iron supplement prescription	1 (1.1)	0 (00.0)	1 (00.6)

Chi-square (χ2) test: P = 0.09

## Discussion

The integral nature of the care proposed by the Brazilian Health Ministry related to the set of actions for providing attention to nuclear or expanded family involves couple and child health care as a whole and is not restricted to primary attention. This objective is achieved via a hierarchically interconnected service network involving several help levels, which guarantee a reference and counter-reference system (1).

This study showed that majority of health professionals were women aged from 30 to 39 years. The study also showed that these health professionals held a graduate degree; had been graduated for >10 years; had been working in the basic health network system for an average of 6 years; were involved in everyday practice, whether medical or nursing; and helped patients on a daily basis in the health centres in Florianopolis, Brazil.

In 2006, the Brazilian Health Ministry published a protocol containing actions to prevent congenital anomalies in the pregestational period. In the same year, the Health Department of Florianopolis, Brazil, also launched a protocol with the same purpose (17).

Although 81.9% of professionals interviewed in this study referred to using some protocol in their practice (with 63.7% of interviewed professionals referring to understand this type of service), less number of activities were aimed to achieve this during the month when these professionals were interviewed. This less number of interventions for the prevention of congenital defects in the 

preconception period indicated a reduced sensitivity to the problem and poor knowledge of the theme described in the protocol. Our data indicate that 48.1% of participants consider his or her knowledge to be of a low level, which is supported by the lack of statistically significant differences between the two groups of professionals. The same happens to the category ‘satisfactory’ knowledge, which reaches the value of 39.4% of the professionals, doctors being the most predominant section, counting on 59.1% when compared to the number of nurses (23.6%) although these values also do not present significantly statistical difference.

Although 39.4% of professionals affirmed that their knowledge was satisfactory, there was a gap between the attitudes of the professionals and the recommendation of the CDC guidelines or the municipal protocol. This gap corroborates the fact that 85.6% professionals mentioned that they did not receive training for preventing congenital defects, a theme that is a part of the municipal protocol.

Among the 14 interventions proposed by the CDC (5), use of folic acid was prescribed by 54.5% of professionals, which was consistent with that reported by Heyes *et al* (14) in their study of gynaecologists and obstetricians involved in primary care. The study by Heyes *et al* (14) evaluated the importance of preconception care by gynaecologists and obstetricians and identified the interventions that they prescribed during their practice. Besides folic acid, Heyes *et al* also identified a relative preoccupation from these professionals with the gestation planning and cigarette dependence, showing little worry about physical activity and environmental exposure (14). 

Interrupting the use of drugs, alcoholic drinks and cigarettes before conception, as recommended by the CDC, is crucial for preventing many anomalies such as foetal alcoholic syndrome that is characterised by impaired neurological development, learning or behavioural deficits, low birth weight leading to cerebral palsy and labial-palatal fissure (18). In this study, these interventions were mentioned as being important by 31.9% of health professionals. In studies by Kitamura *et al* and in Heyes *et al* interruption of smoking and drug use was the most cited intervention by health professionals. Restriction of alcohol consumption was also one of the interventions mentioned as being important by the professionals (19, 14).

Family investigation, although not a part of the 14 interventions recommended by the CDC, was the most referred activity by the health professionals (19.4%). This practice is important because it fosters an aetiological investigation of congenital malformation and informs an individual and his or her family regarding the possibilities of prevention and recurrence of the pathology of a congenital defect (20). It is worth highlighting that such an intervention, referred spontaneously by the professionals in the open-ended question, has higher percentage than some of the items recommended by the CDC.

Some experts indicate that health system policies in Brazil show an insufficient governmental response to actions aimed at preventing congenital defects. Nonetheless, it is worth mentioning the governmental actions aimed at enriching wheat and corn flour with folic acid according to a standardisation proposed by ‘Agência Nacional de Vigilância Sanitária’ (ANVISA) for reducing the rate of neural tube defects without affirming that the government is alienated regarding this problem. Presence of public policies for preventing congenital anomalies has been noticed lately because of relative pressure from health professionals and basic help network for amplifying the theme after congenital defects were established as the first causes of mortality in children aged <1 year for > 10 years.

In summary, this study aimed to investigate the performance of doctors and nurses from the primary care unit in the municipality of Florianopolis, Brazil, related to the prevention of congenital defects in the preconception period. With respect to their knowledge regarding the prevention of congenital defects in the preconception period, it was observed that these professionals recognised the presence of a protocol that subsidises its practice but were unsure of its specific content. With respect to the professionals’ interest in the subject matter, there was a difference between the two groups, with majority of nurses considering themselves as having limited knowledge of the theme while doctors believing that they had satisfactory knowledge, according to their self-evaluation. 

## Note

***This article extracted from Master thesis (Flávia Romariz Ferreira).***

## References

[1] Brasil. Presidência da República. Lei nº 9.263, de 12 de janeiro de 1996. Regula o § 7º do art. 226 da Constituição Federal, que trata do planejamento familiar, estabelece penalidades e dá outras providências. Brasília.

[2] Mumtaz Z, Salway S, Shanner L, Zaman S, Laing L (2012). Addressing disparities in maternal health care in Pakistan: gender, class and exclusion. BMC Pregnancy Childbirth.

[3] Hoke TH, Mackenzie C, Vance G, Boyer B, Canoutas E, Bratt J (2015). Integrating family planning promotion into the work of environmental volunteers: A population, health and environment initiative in Kenya. Int Perspect Sex Reprod Health.

[4] Frey KA, Files JA (2006). Preconception healthcare: what women know and believe. Matern Child Health J.

[5] Klerman LV (2006). Family planning services: an essential component of preconception care. Matern Child Health J.

[6] Cordero JF (2007). Recommendations to improve preconception health and health care [Internet].

[7] Biermann J, Dunlop AL, Brady C, Dubin C, Brann A Jr (2006). Promising practices in preconception care for women at risk for poor health and pregnancy outcomes. Mater Child Health J.

[8] Lu MC, Kotelchuck M, Culhane JF, Hobel CJ, Klerman LV, Thorp JM Jr (2006). Preconception care between pregnancies: the content of internatal care. Mater Child Health J.

[9] Dunlop AL, Jack B, Frey K (2007). National recommendations for preconception care: the essential role of the family physician. J Am Board Fam Med.

[10] (1989). Fundo das Nações Unidas para a Infância. Situação Mundial da Infância.

[11] Duchiade MP, Carvalho ML de, Leal MC (1989). As mortes “em domicílio” de menores de um ano na região metropolitana do Rio de Janeiro em 1986 - um “evento - sentinela” na avaliação dos serviços de saúde. Cad Saude Publica.

[12] Salotti JA, Tennant PW, Windebank K, Rankin J (2015). Langerhans cell histiocytosis in children with congenital anomalies: A population-based record linkage study. Birth Defects Res A Clin Mol Teratol.

[13] Center for Disease Control and Prevention Department of Health and Human Services. Preconception care.

[14] Heyes T, Long S, Mathers N (2004). Preconception care: practice and beliefs of primary care workers. Fam Pract.

[15] Bardin L (1977). Análise de conteúdo Lisboa: Edições.

[16] (2001). Brasil. Ministério da Saúde. Parto, Aborto e Puerpério: Assistência humanizada á mulher . Brasília: Secretaria de Políticas de Saúde. Diretos Reprodutivos, Saúde Materna e Perigestational.

[17] Santa Catarina (2006). Prefeitura municipal de florianópolis secretaria municipal de saúde diretoria de atenção primária gerência de programas estatégicos programa saúde da mulher.

[18] Pollard I (2007). Neuropharmacology of drugs and alcohol in mother and foetus. Semin Fetal Neonatal Med.

[19] Kitamura K, Fetters MD, Ban N (2005). Preconception care by family physicians and general practitioners in Japan. BMC Fam Pract.

[20] Gitsels-van der Wal JT, Martin L, Manniën J, Verhoeven P, Hutton EK, Reinders HS (2015). Antenatal counselling for congenital anomaly tests: Pregnant Muslim Moroccan women׳s preferences. Midwifery.

